# Supplementary material for: Identification of photoperiod-induced specific miRNAs in the adrenal glands of Sunite sheep (Ovis aries)
Source: Front Vet Sci. 2022 Jul 22;9:888207. doi: 10.3389/fvets.2022.888207 (PMC9354845; doi:10.3389/fvets.2022.888207)
Supplement: Supplementary file 1 [file Data_Sheet_1.ZIP › Supplementary file/miRDeep2 file.zip!/miRDeep2 file/Know_Structure/SPLP42A2/oar-mir-30b.pdf]

miRBase precursor : oar-mir-30b
Total read count : 27940
oar-miR-30b read count : 27651
remaining reads : 289

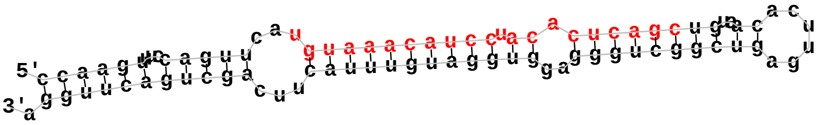

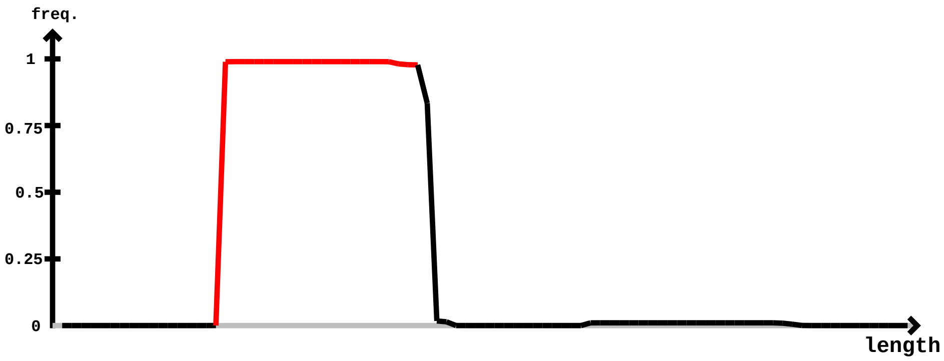


```
                                oar-miR-30b
5'- ccaaguuuccaguucauguaaacauccuacacucagcuguaacacuugagucggcugggagguggauguuuacuucagcugacuugga   -3'    exp
    ((((((...(((((...((((((((((.((.((((((((..((......))))))))))..))))))))))))...))))))))))).    reads      mm         sample
    ..............cauguaaacauccuacacucagcu..................................................    1          0          seq
    ...............auguaaacauccuacacucagcu..................................................    4          0          seq
    ................uguaaacauccuacacu.......................................................    5          0          seq
    ................uguaaacauccuacacA.......................................................    1          1          seq
    ................uguCaacauccuacacu.......................................................    1          1          seq
    ................uguaaacauccuacacuc......................................................    178        0          seq
    ................uguaaacauccuacacuU......................................................    46         1          seq
    ................uguGaacauccuacacuc......................................................    1          1          seq
    ................uguaaacaCccuacacuc......................................................    2          1          seq
    ................uguaaacauccuacacuca.....................................................    87         0          seq
    ................uguaaacauccuacacucag....................................................    22         0          seq
    ................uguaaacauccuacacucagc...................................................    4008       0          seq
    ................uguaaacauccuacacucagcu..................................................    22797      0          seq
    ................uguaaacauccuacacucagcug.................................................    91         0          seq
    ................uguaaacauccuacacucagcugu................................................    390        0          seq
    ................uguaaacauccuacacucagcugua...............................................    2          0          seq
    .................guaaacauccuacacucagcu..................................................    14         0          seq
    .................guaaacauccuacacucagcugu................................................    1          0          seq
    ......................................................cugggagguggauguuuU................    1          1          seq
    ......................................................cugggagguggauguuua................    1          0          seq
    ......................................................cugggagguggauguuuacu..............    38         0          seq
    ......................................................cugggagguggauguuuacuu.............    103        0          seq
    ......................................................cugggagguggauguuuacuuc............    130        0          seq
    ......................................................cugggagguggauguuuacuuca...........    10         0          seq
    .......................................................ugggagguggauguuuacu..............    1          0          seq
    .......................................................ugggagguggauguuuacuu.............    3          0          seq
    .......................................................ugggagguggauguuuacuuc............    2          0          seq
```
